# Primary Hyperparathyroidism Causing Psychosis: A Case Report

**DOI:** 10.7759/cureus.31935

**Published:** 2022-11-27

**Authors:** Aaron Z Meng, Yuyuan Tan, Shao J Ong, Bernard B Wee, Lycia Teo

**Affiliations:** 1 Psychiatry, Ng Teng Fong General Hospital, Singapore, SGP; 2 Radiology, National University Hospital, Singapore, SGP; 3 Radiology, Ng Teng Fong General Hospital, Singapore, SGP

**Keywords:** osteoporosis, paranoia, auditory hallucination, psychotic depression, adult primary hyperparathyroidism, parathyroid adenomas, late-onset psychosis

## Abstract

New-onset psychotic symptoms presenting late in life can be caused by various medical and psychiatric conditions. The index of suspicion for an organic cause for psychotic symptoms in an elderly person should be high, and every presenting patient should undergo a detailed history-taking and evaluation before attributing these symptoms to a primary psychiatric condition. Hyperparathyroidism is one condition that can present with psychiatric symptoms such as low mood and anxiety. While psychiatric symptoms are not uncommon in hyperparathyroidism, acute psychosis is rare. This case report highlights the importance of a thorough evaluation of an elderly person presenting with a new onset of psychosis.

## Introduction

Late-onset psychotic symptoms are not uncommon among older adults. Based on the Well-being of the Singapore Elderly (WiSE) study findings, the prevalence of psychotic symptoms among older adults was estimated at 5.2% [1]. These symptoms may present as a mood disorder (e.g., depression or bipolar disorder), a neurodegenerative disorder (e.g., dementia), an underlying medical condition (e.g., delirium), or a primary psychotic illness such as schizophrenia [2]. In comparison with patients presenting with psychotic symptoms earlier in life, the underlying etiology of late-onset psychotic symptoms tends to be more varied and less commonly due to a primary psychotic illness. In a study based in the United Kingdom, it was reported that the prevalence of a primary psychotic illness such as schizophrenia in late life is 0.1-0.5%, as compared to an overall lifetime prevalence of 1% [3]. With this in mind, it is important to ensure that older adults presenting with late-onset psychotic symptoms are provided with a detailed clinical assessment, including a physical examination, blood investigations, and brain imaging, to determine the underlying cause.

This case report illustrates the case of an elderly lady who presented with acute late-onset psychotic symptoms. Following various medical evaluations, several possible causes of her psychotic symptoms were identified, including hyperparathyroidism. However, her symptoms eventually resolved only after a parathyroidectomy was done.

Hyperparathyroidism is a relatively common endocrine disorder with an estimated prevalence of one to seven per 1000 people. Women are more commonly affected, with a female-to-male ratio of 3:1. The incidence of hyperparathyroidism also tends to increase with age [4].

Hyperparathyroidism is associated with hypercalcemia, which can lead to various complications, commonly referred to as "bones, stones, groans, and moans." "Bone" issues include complications such as bone pain, osteopenia, osteoporosis, and fractures. Kidney stones may also develop and present with renal colic, dysuria, hematuria, and urinary frequency. Other complications of hyperparathyroidism include abdominal symptoms such as nausea, anorexia, reflux, and constipation. Lastly, mental state changes such as fatigue, poor memory, impaired concentration, depression, anxiety, and insomnia may also be present. As the symptoms of hyperparathyroidism can be rather non-specific, delayed or missed diagnoses may occur [5].

It is estimated that one in four people with hyperparathyroidism will experience psychiatric symptoms [6]. Psychiatric symptoms secondary to hyperparathyroidism occur more frequently in the elderly and tend to be more severe than in those in the younger age group [6]. The more common symptoms include cognitive impairment, depressed mood, anxiety, irritability, insomnia, and suicidal thoughts. Depressive symptoms are observed in about 75 % of patients, while psychotic symptoms are much less common at 3% [5,6].

## Case presentation

Ms. A is a 70-year-old East Asian female. She was admitted to our general hospital following an acute change in mental state over a two-week period. Prior to her admission, Ms. A suffered from long-standing lower back pain, for which she regularly took traditional Chinese medications of unknown formulations. She did not have any other chronic medical conditions or past psychiatric illnesses. She also did not have any pre-existing cognitive problems and had been working as a cleaner before her admission.

At the onset of her illness, Ms. A was reported to have a low mood with significant anhedonia and had stopped doing things that used to interest her. With her weight loss, she also experienced insomnia and a loss of appetite. There were no other associated physical symptoms. She did not complain of a headache, giddiness, chest pain, shortness of breath, abdominal discomfort, or urinary symptoms.

Over the course of the subsequent two weeks, Ms. A exhibited increasingly disorganized behavior and psychotic symptoms. She would share stories of her past with others and send text messages to random people on her phone contact list. Her speech was disorganized and illogical, and her family members as well as her colleagues at work found it difficult to understand her. Ms. A also started experiencing auditory hallucinations, reporting that she heard the fan telling her that her son was doing bad things. In addition, she developed persecutory delusions that her daughter-in-law and employers were trying to harm her. For instance, she suspected that her daughter-in-law was spiking her water with worms.

Upon admission to the hospital, Ms. A was observed on a mental state examination to be disoriented and confused. Her mood was depressed, and she appeared preoccupied. Her thought form was disordered, and her speech was irrelevant and tangential. She was observed to have ongoing hallucinations and persecutory delusions in the ward. Her physical examination was unremarkable, with no focal neurological deficits, and her vital signs were stable.

Following her admission, she was referred to a neurologist and psychiatrist for further evaluation. Extensive investigations were then done in view of her late age and her first presentation of acute psychotic symptoms.

Ms. A’s full blood count, serum electrolytes, liver function tests, and thyroid function tests were within the normal range. She had a high corrected serum calcium level of 2.88 mmol/L (reference range 2.10-2.55 mmol/L), a low serum phosphate level of 0.63 mmol/L (reference range 0.74-1.52 mmol/L), and a normal serum magnesium level of 0.67 mmol/L (reference range 0.66-1.07 mmol/L). Her parathyroid hormone level was high at 36.9 pmol/L (reference range 1.6-7.0 pmol/L). Her vitamin D level in her serum was low, at 12 ng/L (the reference range is <20 ng/mL = deficient). She also had a low serum vitamin B12 level of <92 pmol/L (reference range: 138-652 pmol/L) and was positive for anti-parietal cell antibody and anti-intrinsic factor antibody. Her myeloma panel was negative.

There were concerns that the traditional Chinese medication she was taking prior to her admission may contain steroids or metallic toxins, but blood investigations did not support this. Formal toxicological testing could not be done because samples of this Chinese medication were unavailable.

An electroencephalogram showed no focal slowing or epileptiform discharge.

A lumbar puncture was performed, and the analysis of her cerebrospinal fluid (CSF) was unremarkable. There was no biochemical abnormality; CSF cultures were negative; CSF cytology was acellular; and flow cytometry was negative.

Magnetic resonance imaging (MRI) and magnetic resonance angiography (MRA) of her brain demonstrated global cerebral atrophy and chronic small vessel disease, which were within normal limits for her age.

Ms. A’s bone mineral density scan revealed osteoporosis with a hip Z-score of -0.9 SD, a T-score of -2.8 SD, a spine Z score of -3.1 SD, and a T-score of -5.1 SD.

Ms. A was started on an oral atypical antipsychotic medication, Risperidone, while she was undergoing evaluation in the ward. Her vitamin B12 deficiency was treated with daily replacement followed by weekly intramuscular vitamin B12 injections, and she was started on an oral replacement for her vitamin D deficiency. She was also started on subcutaneous calcitonin and intravenous zoledronic acid for the treatment of hypercalcemia and osteoporosis. Her hydration status was optimized with intravenous fluids.

Despite this, her calcium levels became further elevated (3.04 mmol/L), as did her parathyroid hormone levels (41.3 pmol/L). Ms. A also continued to experience psychotic symptoms such as hallucinations and persecutory delusions. She was irritable and agitated, refusing food and medications in the hospital. Oral risperidone was administered in her best interest but did not have a significant effect on her symptoms. As such, she was further observed as an inpatient and underwent further evaluation.

**Figure 1:**
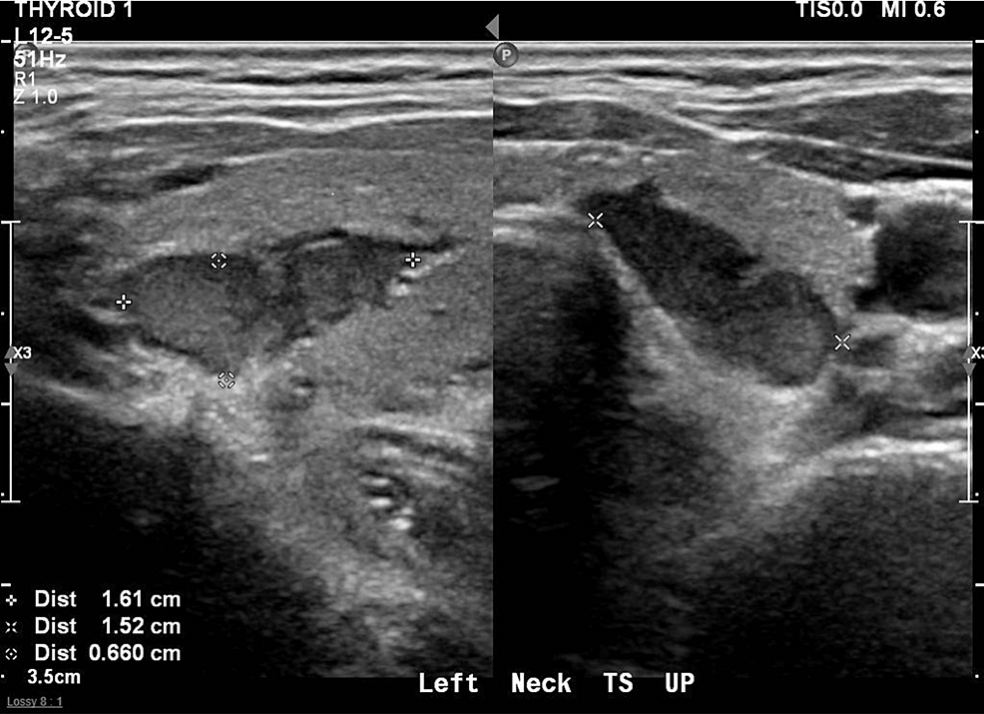
Ultrasound using a linear probe demonstrated a hypoechoic, well-defined, solid lesion at the left upper neck adjacent to the left lobe of the thyroid.

An ultrasound of her thyroid was performed and showed a 1.6 x 1.5 x 0.7 cm hyper-vascular, hypoechoic, solid nodule with well-defined borders at the left upper neck (Figure 1). There was also a 1.9 x 1.5 x 1.2 cm solid-cystic nodule with peripheral vascularity at the left lower thyroid pole. Fine-needle aspiration of the thyroid nodule was done, and the cytology and histology findings were benign.

**Figure 2:**
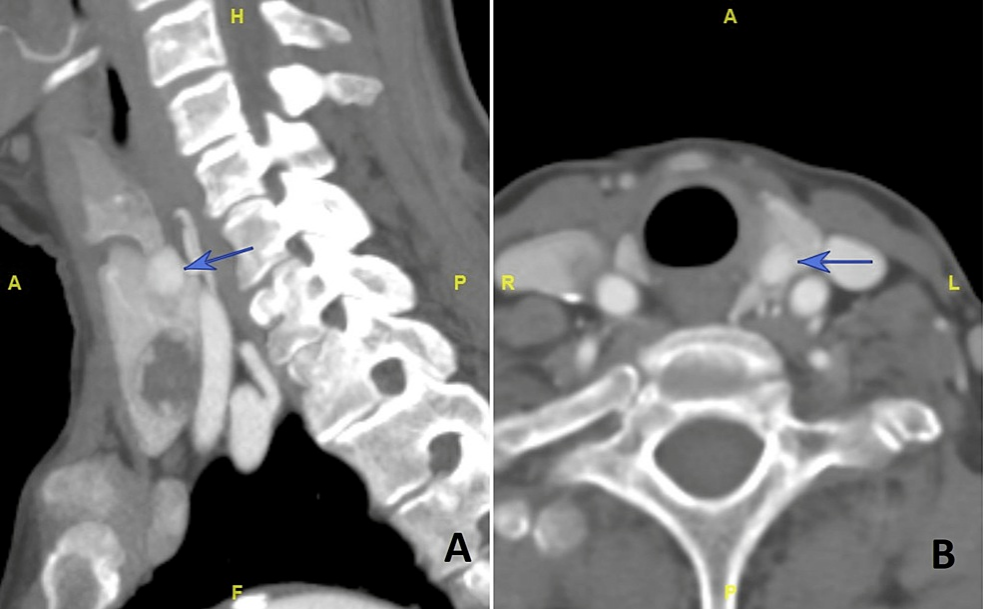
Computed tomography, sagittal (A) and axial (B) imaging, in the arterial phase following the administration of intravenous contrast medium, demonstrated a well-circumscribed arterially enhancing lesion (blue arrow) posterior and medial to the left lobe of the thyroid.

A computed tomography (CT) carotid angiogram showed a corresponding 0.7 x 0.5 x 0.7 cm nodule medial and posterior to the upper pole of the left thyroid gland, with arterial enhancement and washout on the delayed phase, suggestive of a parathyroid adenoma (Figure 2). Another tiny nodule with similar enhancement adjacent to and posterior to the aforementioned nodule measuring about 0.5 x0.2 x0.3 cm was also seen and was postulated to be of parathyroid origin. A mixed solid cystic left lower pole thyroid nodule was also noted.

A consensus from the neurologist, psychiatrist, and endocrinologist was eventually reached that her psychotic symptoms could be secondary to her hyperparathyroidism. Ms. A then underwent a left superior parathyroidectomy, and histology confirmed a parathyroid adenoma.

Ms. A recovered well during the postoperative period. Her electrolyte levels were regularly monitored and replaced when necessary. Her psychotic symptoms also improved markedly following her surgery. She was oriented and no longer thought of herself as disordered. She did not experience any further hallucinations or persecutory ideas. However, it was noted that Ms. A. still had some residual psychomotor slowing and lethargy in the immediate postoperative period.

Ms. A was discharged well and continued on oral risperidone with a dose of 2 mg once at night. She was followed up in the outpatient clinic and was stable, with no further recurrence of her psychotic symptoms. Her low-mood symptoms had also resolved without requiring any antidepressant medication. Furthermore, her post-surgery residual psychomotor slowing and lethargy had abated, and she returned to her premorbid baseline mental state within six weeks of her surgery.

## Discussion

Hypercalcemia can lead to a variety of psychiatric sequelae, ranging from mood and anxiety disorders to cognitive and psychotic symptoms. The onset of symptoms and progression may vary from weeks to months [6]. As psychiatric symptoms are sometimes the only presentation of hypercalcemia, it is prudent for psychiatrists to check for serum calcium levels as part of the evaluation of any new onset of psychiatric symptomatology. This is especially so if the symptoms occur in late life, are atypical in nature, or if the person has specific risk factors for developing hypercalcemia. These risk factors may include a family history of hypercalcemia, renal failure, a known malignancy, or the intake of certain types of medication [7]. If hypercalcemia is found, a referral to an endocrinologist should be made for further evaluation.

Follow-up evaluation of hypercalcemia should include screening for primary hyperparathyroidism, as this is one of the most common causes of hypercalcemia [8]. The index of suspicion for hyperparathyroidism as the underlying cause should also be higher among elderly females. Most cases of primary hyperparathyroidism are caused by parathyroid adenomas (85%) or hyperplasia (15%), with malignancy accounting for less than 1% of reported cases [9]. In these cases, a para-thyroidectomy should be considered as it is a relatively safe treatment option and offers a good chance of symptom resolution [10-13].

Ms. A’s case study has demonstrated the importance of a detailed evaluation for possible underlying medical conditions in an older person presenting with acute late-onset psychotic symptoms. Surgical resection of her underlying parathyroid adenoma and subsequent resolution of her psychiatric symptoms demonstrated that this is a potentially reversible cause of late-onset psychotic symptoms. This is important to recognize, as it will help avoid the unnecessary use of long-term antipsychotic medications and their potential side effects.

Another important learning point from Ms. A’s case study is to recognize that a mildly elevated serum calcium level can lead to significant psychiatric sequelae, such as severe psychotic symptoms. This is consistent with earlier studies demonstrating a poor correlation between serum calcium levels and symptom severity [5, 11, 14]. Regardless of the severity of hypercalcemia, it remains important to consider this as a cause of acute late-onset psychotic symptoms and assess for underlying hyperparathyroidism as a differential diagnosis in these patients.

## Conclusions

Primary hyperparathyroidism is not uncommon. It should always be considered one of the differential diagnoses when evaluating a person with an acute change in mental state, especially in the older age group. This is critical because not only is the condition treatable, but resolving psychiatric symptoms would eliminate the need for long-term antipsychotic use and its potentially harmful effects. 
